# Response: Commentary: Probing Genomic Aspects of the Multi-Host Pathogen *Clostridium perfringens* Reveals Significant Pangenome Diversity, and a Diverse Array of Virulence Factors

**DOI:** 10.3389/fmicb.2018.01857

**Published:** 2018-08-16

**Authors:** Raymond Kiu, Lindsay J. Hall

**Affiliations:** ^1^Gut Microbes and Health Programme, Quadram Institute Bioscience, Norwich Research Park, Norwich, United Kingdom; ^2^Norwich Medical School, University of East Anglia, Norwich Research Park, Norwich, United Kingdom

**Keywords:** pangenome, antimicrobial resistance, *Clostridium perfringens*, genomics, whole genome sequencing, Clostridial infection, exotoxins, pathogen

We firstly would like to express our appreciation for the comments and efforts made by Gohari and Prescott regarding their detailed commentary of our *Clostridium perfringens* Whole Genome Sequencing (WGS) based genomic and phylogenetic study we published in December 2017. We are particularly pleased to note that the authors acknowledge that our “work has contributed significantly to understanding of genomic diversity of this bacterium.”

The aim of our study was to conduct a robust large-scale WGS study on this important pathogen, and to highlight genomic insights to the global scientific community. We believe that WGS could prove extremely useful in exploring new traits in *C. perfringens*, as has been performed in many different bacterial pathogens. We agree with the authors, and indeed highlight this throughout our original study, that alongside genomic-based studies, clinical metadata, epidemiological studies, and phenotypic testing, will be central to determine the impact of genetic variation in *C. perfringens* in the context of human and animal health.

We would first like to respond to the authors commentary suggesting a “clear mistake” or “incorrect conclusion” in that *netB* gene (a gene that encode NetB toxin, which is associated with avian Necrotising Enteritis) has no role in canine haemorrhagic gastroenteritis and foal necrotising enteritis (Keyburn et al., 2008, 2010; Rood et al., 2016). We appreciate the authors have a track record of working in this particular area of *C. perfringens* virulence, as highlighted by citation of their own work throughout the commentary. They highlight that *netB* and *netE* genes (which encodes proteins NetB and NetE respectively) share high sequence identity of 78% amino acid (according to their published work in 2015, 79% amino acid sequence identity; Mehdizadeh Gohari et al., 2015), which may have potentially contributed to our “understandable misinterpretation” of the data. Based on our informatics filtering parameters, we also determined that *netB* and *netE* are highly identical and not distinctive at nucleotide sequence level. This was based on strict double-filtering strategy at 80% identity and E-value of 10^−20^, (routinely applied in WGS studies to infer identical genes; Pearson, 2013; Kiu et al., 2017), and was used as we were undertaking a global *in silico*-based approach to explore a significant number of *C. perfringens* genomes for virulence-associated traits (not solely focused on these two toxin genes). Consequently, we have reanalysed the data and determined that a higher sequence identity threshold at 90% (BLASTn) confirms an absence of the *netB* gene in these NetF-associated genomes. We thank the authors for highlighting the high sequence similarly of these toxin genes, and will factor these parameters in for future studies (Camacho et al., 2009). As we carried out a purely bioinformatic-based study, and are aware of the sensitivity/specificity of different computational pipelines and parameters, we were careful not to come to any definitive “conclusions,” and “suggested” that NetB toxin “might be involved,” thus clarifying our discussion points. Notably, with the advancement in bioinformatics tools, differentiation between highly similar genes may be possible using a “best-match” approach to avoid inaccurate annotations.

To address the question as to whether or not NCTC8503 is an “NE isolate,” we have re-traced the source of this isolate and its sequence data. NCTC8503 is a *C. perfringens* strain isolated by Bennetts (1930) according to the history record shown on the Public Health England NCTC official website^1^ Nairn and Bamford (1967) state “Bennetts (1930) had earlier reported the isolation of *Bacillus welchii* from a bowel lesion in a Black Orpington pullet with intestinal coccidiosis, and he considered that an enterotoxaemia had contributed to the bird's death”; *B. welchii* was later renamed as *C. perfringens*. Furthermore, Williams et al. states “A frequent, although sporadic, poultry clostridiosis (necrotic enteritis [NE]) was first recorded by Bennetts (1930) in Australia,” thus these descriptions indicated a likely link to strain NCTC8503, a *C. perfringens* strain isolated in 1930. We agree that no type D *C. perfringens* strains have been linked with poultry NE. Unfortunately, as NCTC8503 is a historical isolate with no detailed source information recorded, we have been unable to definitively confirm the isolate's origin (Bennetts, 1930; Williams, 2005). However, we can reaffirm that this isolate has not lost the epsilon toxin plasmid during laboratory passage as confirmed by both WGS (Figure 1), and multiplex PCR toxinotyping (Baums et al., 2004; Kiu et al., 2017).

**Figure 1:**
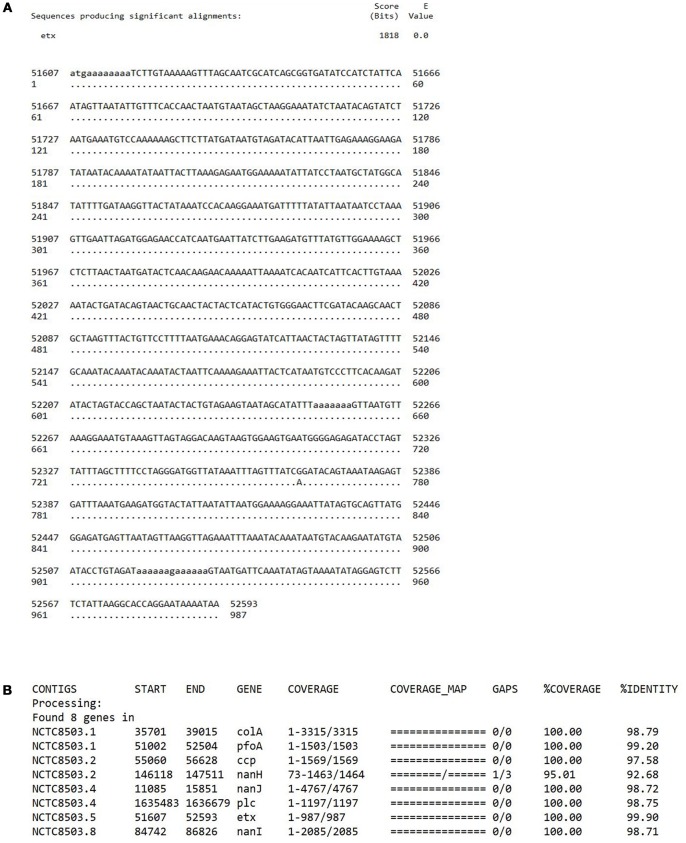
**(A)** A BLASTn sequence alignment performed on a local machine that aligns epsilon toxin gene *etx* (NCBI accession: M95206.1) with PacBio-sequenced NCTC8503 previously assembled genome (ENA accession: SAMEA3879480; contig 5: nucleotide position 51607-52593). Only a point mutation at position 762 is detected with 100% coverage and > 99% sequence identity. **(B)** Toxin profile of NCTC8503 isolate, performed via sequence similarity search (“best-match” approach) pipeline ABRicate (https://github.com/tseemann/abricate; BLASTn-based tool) that confirmed the presence of *etx* gene in NCTC8503 genome with 99.90% identity (at 100% coverage).

The “extreme” level of pangenome (12.6% core genes) in *C. perfringens* has not been widely reported to date (McInerney et al., 2017). However, as stated by the authors a previous study carried out in 2010 in *Escherichia coli*, which analysed 53 genomes, indicated 11% core genes (Lukjancenko et al., 2010). Notably, although these 2 bacteria can colonise the GI tract, they are fundamentally different regarding their oxygen sensitivity and ability to form spores. As described by Lukjanenko et al., analysis of 53 genomes (not 61 genomes as claimed) identified 1,472 core genes out of 13,296 genes in the pangenome (11%), which is lower than the 12.6% reported in our genomic *C. perfringens* analysis, although we note some differences in approaches used. More recently a pangenome study based on 228 *E. coli* genomes identified 23.8% core genes (2,722 core genes in 11,401 gene families) (McNally et al., 2016), and using web-based tool panX pangenome analysis^2^ and databases (Ding et al., 2018), based on the same cutoff as our study indicated 13% of core genes, based on analysis of 307 *E. coli* genomes (as of May 2018). We reiterate that our “extreme” observation for spore-forming Gram-positive *C. perfringens* is a rare trait, and is therefore of interest to the wider research community. We agree with their statement “describing a species as having extreme variation depends very much to what it is being compared,” and furthermore we would emphasise that prediction of genetic diversity will likely vary due to different factors including sampling bias, number of strains selected, and parameters used during informatics analysis, thus impacting diversity measurements. In this study we compared *C. perfringens* with *Clostridium difficile* (30.3%, which has been reclassified as *Clostridioides difficile*), a closer relative of *C. perfringens*, and *Streptococcus pneumoniae* (46.5%), *Salmonella enterica* (16%), and *Klebsiella pneumoniae* (26%) (Lawson et al., 2016; Kiu et al., 2017).

We agree that there is currently no definitive pathogenicity link with *C. perfringens* prophages, although previous studies have indicated they enhance sporulation, which would be expected to enhance transmission, and can be viewed as a virulence trait (Stewart and Johnson, 1977). Notably, in the closely related pathogen *C. difficile*, bacteriophages are linked to toxin-secretion (Goh et al., 2005). Consequently, we speculate that *C. perfringens* phages contribute to their virulence, which could be confirmed in future studies.

We hope that our commentary provides clarification and context and we look forward to wider discussion with all investigators in the *C. perfringens* field.

## Author contributions

RK and LH co-wrote the manuscript. RK performed the bioinformatics analysis.

### Conflict of interest statement

The authors declare that the research was conducted in the absence of any commercial or financial relationships that could be construed as a potential conflict of interest.

## Footnotes

^1^https://www.phe-culturecollections.org.uk/

^2^http://pangenome.tuebingen.mpg.de/
